# Pharmacists’ views on barriers and enablers to the implementation of advanced pharmacist prescribing in Scotland: a qualitative study using normalisation process theory

**DOI:** 10.1007/s11096-025-02021-y

**Published:** 2025-10-06

**Authors:** Mairi-Anne McLean, Paul Forsyth, Anne C. Boyter

**Affiliations:** 1 NHS Greater Glasgow and Clyde, Glasgow, UK; 2 University of Strathclyde, Glasgow, UK; 3 Golden Jubilee National Hospital, Clydebank, UK

**Keywords:** Advanced practice, Pharmacy, Prescribing, Normalisation Process Theory

## Abstract

**Introduction:**

Healthcare systems need more professionals able to deliver autonomous holistic advanced care. Exploring pharmacists’ views on the implementation of advanced pharmacist prescribing (i.e. prescribing autonomously in complex, uncertain, or higher-risk situations) will allow stakeholders to reflect on possible changes that may embed and sustain this work.

**Aim:**

To explore pharmacists’ views on barriers and enablers to the implementation of advanced pharmacist prescribing in Scotland.

**Method:**

Semi-structured one-to-one interviews were conducted with National Health Service employed pharmacists from across Scotland, recruited via professional networks using purposive sampling to ensure a range of professional backgrounds, geography and prescribing activity. Interviews were developed and analysed using Normalisation Process Theory (NPT), a sociological framework for understanding how new practices become embedded in routine healthcare and analysed using a hybrid deductive/inductive thematic framework analysis approach.

**Results:**

Thirteen pharmacists from across Scotland participated in the study. Barriers and enablers to advanced pharmacist prescribing were identified in all NPT constructs (Coherence, Cognitive Participation, Collective Action, and Reflexive Monitoring). In responses relating to the Coherence construct, enablers included individual understanding of how advanced pharmacist prescribing differs from other advanced pharmacist tasks, as well as the role of an advanced pharmacist prescriber. Lack of shared understanding emerged as a barrier. Cognitive Participation identified barriers including lack of appropriate roles and training as well as a lack of infrastructure to support advanced pharmacist prescribing. In Collective Action, barriers included lack of confidence in consistent delivery of advanced pharmacist prescribing and resource constraints. Reflexive Monitoring revealed strong individual belief in advanced pharmacist prescribing, but barriers included a lack of multidisciplinary evaluation and a need for service reconfiguration.

**Conclusion:**

While individual pharmacists were found to be committed to advanced pharmacist prescribing, widespread implementation is hindered by a lack of shared understanding, inconsistent role structures, and limited strategic alignment. These findings offer valuable insight for those with an interest in embedding and sustaining advanced pharmacist prescribing.

**Supplementary Information:**

The online version contains supplementary material available at 10.1007/s11096-025-02021-y.

## Impact statements

- The results of this study provide stakeholders with an insight on barriers and enablers when seeking to embed and sustain delivery of advanced pharmacist prescribing.
- Future work with senior pharmacy leaders and educators should focus on addressing barriers to advanced-level prescribing, including the lack of shared understanding and limited engagement with prescribing at advanced-level.

## Introduction

The pressure of aging populations, advances in healthcare technologies, increasing expectations, and fiscal challenges have accelerated the evolution of non-medical healthcare roles, including the introduction of pharmacist prescribing in several countries. The United Kingdom (UK), United States, Canada, Australia, Poland, Switzerland, and Denmark [1] have introduced pharmacist independent prescribing to improve healthcare provision [2, 3]. As other countries seek strategies to manage workforce challenges and increase access to healthcare, some may consider legalising pharmacist prescribing where not yet available [4], presenting opportunities to explore what factors influence the success of pharmacist prescribing [2, 5–14].

Models of pharmacist prescribing include supplementary (within agreed patient-specific plan), collaborative (shared care where the physician retains primary responsibility), dependent (under physician direction) and independent (prescribing autonomously) [15], with independent prescribing allowing pharmacists to prescribe with the greatest level of freedom and accountability [3]. From 2026, pharmacists will be able to independently prescribe from the point of registration, following implementation of the UK pharmacy regulator’s Standards for the Initial Education and Training of Pharmacists [16].

In the UK, foundation-level independent prescribing pharmacists are able to autonomously apply evidence-based clinical knowledge when making routine prescribing decisions [17]. As pharmacists progress through their careers, they may develop additional knowledge and skills to work at a higher, more advanced level of practice. To support UK pharmacists, the Royal Pharmaceutical Society (RPS) has introduced the Core Advanced Pharmacist Curriculum which outlines competencies [18] across four domains—Clinical Practice, Leadership and Management, Education, and Research—often referred to as the “four pillars” [18]. These pillars align with international frameworks such as the FIP Global Advanced Development Framework [19] and reflect the multi-domain approach to advanced practice. Credentialing at advanced level involves submitting a portfolio of evidence mapped to practice outcomes to evidence that pharmacists can provide holistic, autonomous care for patients with increasingly complex needs, while managing risk and uncertainty, and/or in cases where evidence is lacking [18]. They can use a range of assessment methods, including clinical examination and other skills, adapting both these and communication style based on the needs of the patient [18].

Recent research in the UK found a lack of consensus on whether pharmacists working towards the advanced career stage must be independent prescribers [20]. This is at odds with the RPS Core Advanced Curriculum which states that “pharmacists organically moving through Core Advanced will be prescribers going forward” and that “it is very difficult to imagine how an individual would be able to demonstrate the autonomy required in the [Core Advanced] clinical domains without being able to prescribe”[18]. As key pharmacy stakeholders advocate for advanced-level practice [21, 22] and independent prescribing [16, 23–25], it is important to understand workforce views on the implementation of advanced pharmacist prescribing. Normalisation Process Theory (NPT) is a sociological model designed to provide insight into the implementation of complex healthcare interventions, such as the implementation of advanced pharmacist prescribing. NPT comprises four constructs—Coherence, Cognitive Participation, Collective Action, and Reflexive Monitoring that help explain how individuals make sense of, engage with, enact, and appraise new practices [26].

## Aim

The aim of this study was to explore pharmacists’ views on barriers and enablers to the implementation of advanced pharmacist prescribing in Scotland.

## Method

### Study setting

The study was set in the National Health Service (NHS) in Scotland. NHS Scotland consists of 14 regional NHS Boards, delivering frontline healthcare services, and eight Special NHS Boards, delivering a range of specialist and national services. The NHS is funded through taxation and is, for most services, free at the point of delivery [27]. Scotland has a population of around 5.5 million. Responsibility for the NHS in Scotland is a devolved matter and legislation rests with the Scottish Government.

### Sampling

Eligible participants were registered pharmacists who were directly employed by a regional NHS Board, and/or one of the four Special NHS Boards where pharmacists are engaged in patient-facing or educational activities—NHS Education for Scotland, NHS 24, Golden Jubilee University National Hospital and State Hospitals Board for Scotland.

The purposive sampling strategy was intentionally broad to capture a range of perspectives from those close to advanced pharmacist prescribing, not only those who have formally achieved advanced status, as implementation relies on broad understanding across a range of pharmacy stakeholders. Participants were selected to ensure at least two participants from each of the following categories:

- line managers and non-line managers
- working predominantly in rural areas and in urban areas,
- educationalists (those with an educational component in their role of greater than or equal to 25%) and non-educationalists
- active independent prescribers and non-active independent prescribers

The sample was not intended to be statistically representative.

### Recruitment

An invitation to participate was disseminated by email in March 2023 through professional networks (Directors of Pharmacy, National Acute Pharmacy group, and the Scottish Practice Pharmacist and Prescribing Advisers Association). These networks would reach all eligible pharmacists. No reminder email was required as initial response rate was sufficient. Participants were provided with a link to a survey which contained participant information, consent form, demographics survey and requested an email address from interested participants. Demographic data collected included gender, ethnicity, years registered, independent prescriber status, primary work setting, line manager status, and whether their role included an educational component [29]. Microsoft Meet® was used to allow participants to schedule a time for their interview. An initial round of ten interviews was planned, followed by analysis. Subsequently, three additional interviews would be conducted and analysed. If no new themes emerged, data saturation would be considered achieved. If new themes emerged, three further interviews would be undertaken and analysed, repeating until no new themes emerged [28].

### Data collection and handling

A semi-structured one-to-one interview schedule was developed using NPT [26] and one key publication [29]. Questions were based around the constructs of NPT [26] (See supplementary files). The interview schedule was adapted following piloting to improve clarity and alignment with NPT. Pilot interviews were excluded from the study. The final interview schedule was agreed by the authors. Interviews took place in April and May 2023, using Microsoft Teams®. At interview outset, verbal consent for interview and video recording was reconfirmed (MMcL). Interviews were video-recorded and subsequently transcribed verbatim (MMcL). Transcripts were reviewed for accuracy, anonymised and were not returned to participants for comment. All data relating to the study were stored securely on the University of Strathclyde network.

### Data analysis

Two coders were involved in data analysis, one female (MMcL) and one male (PF), both pharmacists with 21–26 years of experience within primary care, secondary care and community pharmacy. A hybrid deductive/inductive thematic framework analysis approach was used. Sixteen deductive codes were defined a priori from NPT. Primary analysis was undertaken by the lead researcher (MMcL) following familiarisation with the initial data set. Transcripts were first coded deductively using Nvivo^©^ software, with data sorted into the components of NPT. An inductive review was then conducted to identify any additional themes. The second coder familiarised themselves with the first three transcripts, and both coders met to refine the coding approach in consultation with academic supervisors (PF and ACB). Minor changes to the approach were agreed. The lead researcher then adapted the coding of the first three transcripts and applied the agreed deductive approach to the remaining transcripts.

### Ethics approval

Ethics review was conducted by the Strathclyde Institute of Pharmacy and Biomedical Sciences, University of Strathclyde, on 14th March 2023. The department determined that full university ethics approval was not required, as the project was classified as a service evaluation.

## Results

Forty-nine pharmacists consented to participate. Thirteen pharmacists were interviewed (Table 1). Interviews lasted between 34 and 71 min. Participants predominantly worked in an urban work setting (n = 11), with a small representation of rurally based pharmacists (n = 2). Most participants were line managers (n = 10), female (n = 9), and did not have a significant educational component to their role (n = 8). The median time qualified was 18 years. The participants were a split of active prescribers (n = 7) and non-prescribers (n = 6).

**Table 1:** NPT participant demographics

	NPT Interviews (N = 13)
	n = (%)
*Gender*	
Female	9 (69%)
Male	3 (23%)
Non-binary	1 (8%)
*Ethnicity*	
White British	11 (84%)
White other	1 (8%)
Prefer not to say	1 (8%)
*Years Registered*	
Median (IQR1, IQR3)	18 (9.25,27)
*Active Independent prescriber?*	
Yes	7 (54%)
No	6 (46%)
Primary work setting*	
Rural	2 (15%)
Urban	11 (84%)
Line Manager of AfC** Band 7 pharmacist or above?	
Yes	10 (77%)
No	3 (23%)
*Does your role have a significant pharmacist education and training component (around 25% or more of your role)?*	
Yes	5 (38%)
No	8 (62%)

*Participants self-identified as working in predominantly rural or urban setting

**Agenda for Change is the main pay system for staff in the NHS, except doctors, dentists and senior managers. Abbreviated to AfC and also known as NHS Terms and Conditions of Service

**Fig. 1:**
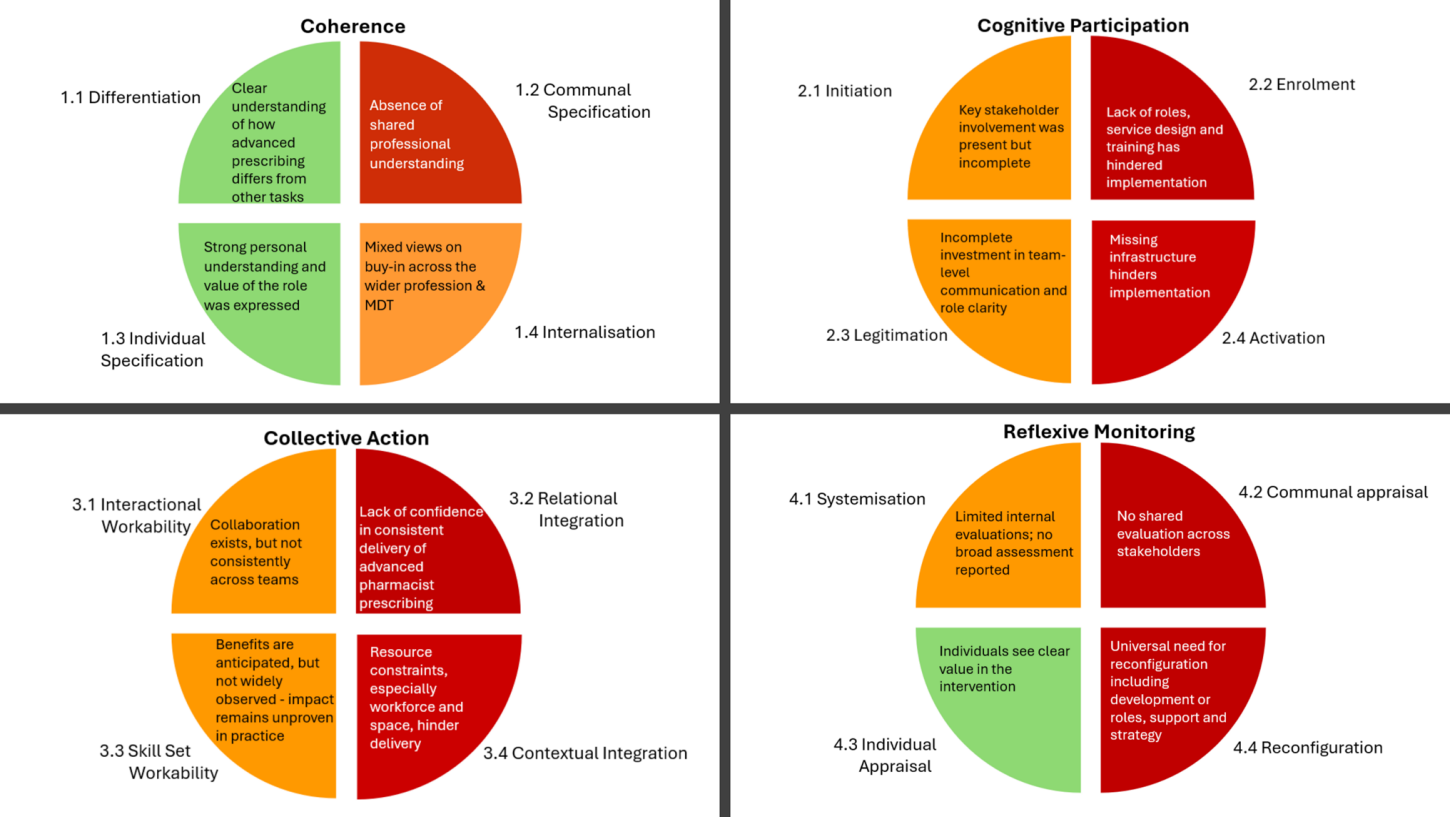
Red—Clear Barrier - The component was absent or insufficient and is a barrier to the intervention. Orange—Ambiguous Presence - The component’s presence was unclear or inconsistently described. Green—Clear Enabler - The component was clearly present and is an enabler for the intervention

Deductive analysis segmented data into the four constructs and 16 components of NPT. No inductive themes emerged. Researchers concluded that data saturation had been achieved when no new themes emerged in the three interviews following the initial ten. A narrative of the results is presented below along with illustrative quotations. The main findings are summarised visually in Fig. 1, which presents the barriers and enablers to implementation mapped to the four constructs of NPT.

### Construct 1–coherence

Responses relating to the Coherence construct (i.e. how people make sense of an intervention) of NPT, showed a strong understanding of how prescribing differs from other advanced pharmacist tasks. This differentiation enables implementation, as pharmacists can define the purpose and value of advanced pharmacist prescribing.

*“I think it’s a different mind-set…quite often you’re having to make a decision where there is no clear right or wrong answer…you have to be able to manage risk and uncertainty”* Participant 11—Urban, non-prescribing, line manager, non-educationalist.

When asked if there was a shared understanding amongst pharmacists on the aims, objectives, and expected benefits of advanced pharmacist prescribing, a few felt there may be understanding in some pharmacists, however all participants raised concerns over broad conceptual misalignment.

*“No would be my basic answer because there’s so much disparity within the profession just now”* Participant 10—Rural, prescribing, non-line manager, educationalist.

*“No, I think that the level of understanding kind of probably peters out above sort of [middle management level]”* Participant 6—Urban, prescribing, line manager, non-educationalist.

Participants all expressed a personal understanding of advanced pharmacist prescribing. They felt they understood the role and viewed it positively, anticipating beneficial impacts on service efficiency, professional development, workforce utilisation, and patient care.

*“I think probably I would say there is a significant impact on service delivery from delivering advanced pharmacist prescribing”* Participant 6—Urban, prescribing, line manager, non-educationalist.

While there was a strong sense of personal engagement from most participants, a lack of engagement was described by a minority of participants and some participants stated that they observed a lack of engagement from others. Lack of universal engagement with advanced pharmacist prescribing specifically as part of a four-pillar RPS framework was mentioned by several participants.

[When asked about stakeholder involvement in the development of advanced pharmacist prescribing]. *“…. we’ve created this lovely career framework, but there’s not really buy-in at the top … [and] across the MDT [Multi-Disciplinary Team]”*.Participant 9—Urban, non-prescribing, line manager, non-educationalist.

*“I think one of the reasons maybe that people are against all of the pharmacists being prescribers to do advanced is the fact that then there is not equity. So, you are saying that advanced is a club that only prescribers can go into”.* Participant 7—Urban, prescribing, line manager, educationalist.

### Construct 2–cognitive participation

The Cognitive Participation construct (i.e. how people engage with an intervention) of NPT identified a common view that there had been incomplete stakeholder involvement in building a community of practice around advanced pharmacist prescribing.

*“I think it’s quite pharmacist heavy, I’m not sure we’ve thought about what the wider MDT is looking for from us.”* Participant 5—Urban, non-prescribing, non-line manager, non-educationalist.

*“I didn’t hear patients, and, you know, medical staff, I didn’t hear them being involved.”* Participant 13—Urban, non-prescribing, line manager, educationalist.

Most participants felt that the required changes to roles had not been made, preventing pharmacists from collectively contributing to the work. This was seen as a barrier to effective implementation.

*“But actually, the job role doesn’t have four pillar working within it, which is then a bit of a challenge if you’re expecting somebody to be credentialed to that [advanced level] but you’re not actually expecting them to work to that [level of clinical practice] within the job, it’s a bit strange.”* Participant 2—Urban, prescribing, line manager, educationalist.

Opinions varied on whether significant efforts had been made to ensure that teams had had sufficient communication around advanced pharmacist prescribing, including clarity on the role of advanced pharmacist prescribers.

*“I suppose we’ve been quite vocal about what our team [of advanced pharmacist prescribers] does and how it operates, and the kind of job planning process really let us show people in a really visual way as well.”* Participant 12—Rural, prescribing, non-line manager, educationalist.

*“So, I have not done much yet. We’re at a very early stage here but it’s absolutely a priority… ask me the same question again in six months and I will give you a very different answer.”* Participant 9—Urban, non-prescribing, line manager, non-educationalist.

Participants identified three key actions—(1) Leadership, vision, and strategy; (2) Workforce development; and (3) Financial investment—required to embed and sustain advanced pharmacist prescribing. Collectively, this can be characterised as “missing infrastructure” which hinders implementation.

*“I think you need the pharmacy support workers to free up the techs, the techs to free up the pharmacists and the pharmacists to free up the advanced pharmacists”* Participant 8—Urban, non-prescribing, line manager, non-educationalist.

*“If I’m wholly honest, it needs to be led by our [senior pharmacy leaders]”* Participant 9—Urban, non-prescribing, line manager, non-educationalist.

### Construct 3–collective action

Responses relating to Collective Action construct (i.e. how people enact an intervention) identified mixed views on how well staff collaborated to deliver advanced pharmacist prescribing.

*“The wider team really do understand our development needs and are really keen to support our development of advanced practice”* Participant 1—Urban, prescribing, line manager, non-educationalist.

*“I would like to say that you see teams working together to deliver [advanced] pharmacist prescribing but I think, at the moment, we are not necessarily all working together”* Participant 2—Urban, prescribing, line manager, educationalist.

While a few participants described isolated examples of delivery, participants all described a lack of confidence that advanced pharmacist prescribing was consistently delivered.

*“Its success is kind of a bit haphazard rather than an overarching strategy”* Participant 7—Urban, prescribing, line manager, educationalist.

There was a strong view that advanced pharmacist prescribing had the potential to improve team efficiency and better utilise pharmacist expertise, however, these benefits were largely anticipated rather than widely observed in practice.

*“We’re taking on GP [general practitioner] workload, ANP [advanced nurse practitioner] workload when we are running advanced clinics or we’re taking heart failure work away from outpatient clinics”* Participant 4—Urban, prescribing, line manager, non-educationalist.

Most participants thought that unmet resource needs were a barrier to implementation of advanced pharmacist prescribing, most notably requirements for more human resource and clinic space.

*“One of the most difficult things for us has been getting clinic space”* Participant 1—Urban, prescribing, line manager, non-educationalist.

### Construct 4–reflexive monitoring

Responses to questions exploring the Reflexive Monitoring construct (i.e. how people appraise an intervention) of NPT were mixed. While a small number of participants felt that there was evaluation, in the form of small-scale internal projects within individual pharmacy teams i.e. not broad profession wide evaluation, others were not aware of any evaluation of advanced pharmacist prescribing. Most respondents felt that there had been no evaluation undertaken outside the profession.

*“I’m not entirely convinced that groups outside pharmacy have been involved in evaluating”* Participant 1—Urban, prescribing, line manager, non-educationalist.

Most felt that they, as individuals, were supportive of the continued development of advanced pharmacist prescribing, however all participants felt that reconfiguration of existing practices, structures, and processes was required to address barriers to the implementation of advanced pharmacist prescribing.

*“We need to have people prescribing full stop and at that point then working towards advanced”* Participant 2—Urban, prescribing, line manager, educationalist.

The lack of appropriate protected roles was identified as the greatest barrier. Better support, strategy, workforce review and reaching a shared understanding of advanced pharmacist prescribing were also seen as necessary to embed delivery.

*“What are we [pharmacists] willing to give up? Because you can’t do everything. You can’t be a specialist and be doing everything. Who is the right person to be doing those tasks?* Participant 2—Urban, prescribing, line manager, educationalist.

*“It* [advanced pharmacist prescribing] *needs to be embedded in job descriptions and job roles, it needs to be defined, and everybody needs to be on board”* Participant 5—Urban, non-prescribing, non-line manager, non-educationalist.

## Discussion

### Summary of key findings

This study aimed to explore pharmacists’ views on barriers and enablers to the implementation of advanced pharmacist prescribing. Pharmacists recognised its potential to improve service delivery, professional development, and patient care and expressed strong personal support for advanced pharmacist prescribing which is an important enabler. However, there were also strong views that implementation was hindered by barriers including lack of understanding, insufficient stakeholder engagement, unmet resource needs, and the absence of appropriate roles.

### Strengths and weaknesses

NPT is a robust model which is frequently used in qualitative healthcare research [30], providing a strong theoretical underpinning to our results. The purposive sampling strategy ensured participant representation from key groups. The interview schedule was piloted and refined before use, ensuring clarity and relevance of questions. Data saturation was achieved and confirmed after 13 interviews, supporting the completeness and credibility of the findings. Coding was conducted by an experienced pharmacist and checked by a second coder, both with substantial clinical experience, with coding decisions subject to supervisory oversight. Using a hybrid deductive/inductive thematic framework analysis approach ensured that all possible themes were captured. The study context and participant characteristics are described in detail, supporting transferability for readers in other settings.

The sample size of the study was small, however saturation was achieved. All participants were NHS employed pharmacists, which may limit the transferability of findings to other sectors (e.g. community pharmacy, academia etc.). Similarly, participants were also from one country only (i.e. Scotland). Although some changes to practice may have occurred since interviews took place, the findings are likely to be largely relevant at time of publication.

### Interpretation

This study found individuals were strongly committed to the development of advanced pharmacist prescribing. This was seen in both an understanding of and belief in the role of the advanced pharmacist prescriber. This is in line with international evolution of non-medical prescribing roles [1, 18, 19, 23, 31, 32] and with findings that pharmacists, in other settings and countries, commonly express a belief in the value of prescribing and see it as a positive contribution [34] to patient care [12, 33, 34]. Participants described that across the pharmacy profession, there was no shared understanding of advanced pharmacist prescribing. Participants expressed concern that senior pharmacy leaders did not demonstrate understanding of the concept and purpose of advanced pharmacist prescribing. Lack of shared understanding of the role and responsibilities of pharmacists in outpatient clinics has also been found to be a barrier to service provision [33] and others have identified a need to build stakeholder understanding of the role and purpose of pharmacist prescribers within pharmacy and beyond [2, 34]. This lack of understanding is not unique to our setting, similar challenges the implementation of pharmacist prescribing have been reported internationally, including in Canada, Australia, and elsewhere in the UK [2, 13].

Lack of understanding of advanced roles is widely recognised as a barrier to the successful implementation of advanced practice and contributes to challenges around professional identity [35–37], which can further hinder implementation efforts [16, 18, 24–26, 39–42]. Professional identity inconsistency where pharmacists do not uniformly see themselves as clinicians or as part of a distinct professional group, limits their confidence, autonomy, and ability to deliver clinical roles [38–40]. Despite the strong strategic direction towards pharmacist independent prescribing [16, 23–25, 41], and prescribing competencies forming an integral part of the RPS Core Advanced Curriculum [18], participants expressed a view that prescribing is not universally accepted as an essential part of advanced practice.

Participants described requirements to embed and sustain advanced pharmacist prescribing, namely (1) Leadership, vision, and strategy; (2) Workforce development; and (3) Financial investment. Previous findings of inadequate support from organisations, leaders and teams [2, 12] directly correlate with this study, highlighting the opportunity for strategic leadership to address this barrier. There needs to be clear leadership on role development as research has found confusion over who is responsible for this task [33]. Investment in supporting and training the workforce is also essential to deliver the vision of pharmacist prescribing at all levels [2, 33, 34, 42]. Research drawn from multiple countries [2] found that inadequate training was the most frequently cited barrier to pharmacist prescribing, highlighting the need for support to build prescriber competence. Lack of funding is also a barrier [2, 42] that must be addressed for prescribing to flourish.

Achieving consistent and sustainable implementation of advanced pharmacist prescribing requires a coordinated, profession-wide approach. There is a need to strengthen collaboration across the profession and beyond to develop the infrastructure and support required to drive consistency in service delivery and ensure the appropriate use of skill mix to support effective and sustainable implementation. Participants in this study described a lack of shared culture and consistent approaches to service delivery. Bailey et al. [33] found consistent care delivery came from roles that were embedded into standard patient treatment pathways. This study found a belief that skills mix was not yet correct to support consistent delivery. Less complex or administrative tasks have been effectively delegated to foundation pharmacists or technical staff [33, 43], however this can only happen when teams have the right mix of roles and competencies to allow pharmacists to focus on prescribing tasks. Participants in this study felt that, if collaboration could be enacted to support delivery as intended, advanced pharmacist prescribing had the potential to further develop the pharmacy skill set and improve service delivery and capacity in the multidisciplinary team. This belief aligns with global advanced practice strategy [21, 22] and highlights the need for senior pharmacy leaders to recognise its strategic value and actively support its implementation.

Participants expressed a need for wider appraisal of the benefits of advanced pharmacist prescribing, working in collaboration with non-pharmacist stakeholders. Evidence of value is an important enabling factor for advanced practice roles [44]. Despite research being recognised as one of the pillars of advanced practice [10, 40], this pillar is not fully embraced in pharmacy [45]. Some small-scale evaluation of advanced pharmacist prescribing within pharmacy was discussed but most participants felt more evaluation of its impact was required. This lack of awareness of formal evaluation may reflect a wider barrier to research itself within pharmacy due to lack of confidence, skills and support to engage in research [46]. There was strong individual support for the continued development of advanced pharmacist prescribing but a clear need to reconfigure existing practices, structures, and evaluation processes to better support its implementation. To fully realise the potential benefits of advanced pharmacist prescribing, senior leaders and educators are encouraged to work together to address barriers and promote enablers.

### Further research

Further research should explore how collective leadership and role clarity can be strengthened to support implementation. Despite clear barriers and broad support, progress remains uneven.

Further research into how to build on the understanding and collaboration in relation to the implementation of advanced pharmacist prescribing would be of value to those seeking to implement advanced pharmacist prescribing.

Additionally, further research should explore how the lack of shared understanding of advanced pharmacist prescribing combined with inconsistent role definitions contribute to ongoing challenges in pharmacists’ professional identity. Investigating how professional identity is shaped, reinforced, and affects these issues may also help inform strategies to support role clarity and professional vision.

## Conclusion

This study highlights a clear commitment among pharmacists to the development of advanced prescribing roles. However, the findings suggest that implementation is hindered by systemic barriers, including a lack of shared understanding, inconsistent role structures, and limited strategic alignment. Embedding advanced pharmacist prescribing will require more than individual enthusiasm; it demands clear leadership, a workforce development strategy, and financial investment. These findings provide a strong foundation for future work aimed at strengthening the infrastructure and professional identity needed to support pharmacists in advanced prescribing roles.

## Supplementary Information

Below is the link to the electronic supplementary material.

Supplementary file 1 (PDF 253 KB)

## Data Availability

The participants of this study did not give written consent for their data to be shared publicly, so due to the sensitive nature of the research, supporting data is not available.
