# Supplementary material for: Pharmacists’ views on barriers and enablers to the implementation of advanced pharmacist prescribing in Scotland: a qualitative study using normalisation process theory
Source: Int J Clin Pharm. 2025 Oct 6;48(2):534–43. doi: 10.1007/s11096-025-02021-y (PMC12992344; doi:10.1007/s11096-025-02021-y)
Supplement: Supplementary file 1 — (PDF 253 KB) [file 11096_2025_2021_MOESM1_ESM.pdf]

Supplementary Files

Pharmacists' views on the barriers and enablers to implementation of advanced pharmacist prescribing in ~~:~~Scotland: a qualitative study using Normalisation Process Theory

Interview Questions

Question	Prompts	Notes
**Coherence**<br>*Coherence refers to the "sense making work" carried out by individuals and collectively within organisations when implementing a new practice, understanding the practice being a pre-requisite to success.*		
**Differentiation** – how a set of practices and their objects are different from each other		
1. Can you describe for me how (if at all) prescribing differs from other activities that pharmacists at the advanced career stage may undertake?	Prompt activities including: making a recommendation, educating, facilitating.<br>Prompt the differences in skills, tasks, responsibilities, training needed, time	
**Individual specification** – strong individual understanding of the work		
2. To what extent do you think being a prescriber affects the impact of a pharmacist working at the advanced career stage?	Is prescribing an integral part of all job roles at the advanced career stage?<br>What difference does prescribing make to patients/colleagues, pharmacists, rest of MDT, organisation?	
**Communal specification** ~~-~~- shared understanding of the aims, objectives, and expected benefits		
3. Do you think that the profession have a shared understanding of the aims, objectives, and expected impact of prescribing at the advanced pharmacist career stage?	Do you think the whole profession agree one the impact that prescribing [in the advanced pharmacist career stage] has on patient care?	

**Internalisation** - are individuals bought into this work?		
4. Do you think prescribing [in the advanced pharmacist career stage] is a priority for pharmacists at all levels of seniority and grade?		
**Cognitive Participation**<br>*Refers to the "relational work" carried out to "build and sustain a community of practice" around the intervention, including the involvement of key stakeholders to drive the intervention forwards.*		
**Initiation** - involvement in making the intervention work		
5. To what extent (if at all) have key stakeholders been involved in the development of pharmacist prescribing in the advanced career stage?	Who are the key people who are delivering this strategy?<br><br>How have you been involved in developing the strategy, if at all?	
**Enrolment** - Participants may need to organize or reorganis~~z~~e themselves and others in order to collectively contribute to the work		
6. Can you describe any changes that you have made to your role in order to ensure that pharmacist prescribing in the advanced career stage is successfully delivered?	Prompt: uptake, enthusiasm, impact of involvement on workload, skills etc.	
**Legitimation** - the work of ensuring that other participants believe it is right for them to be involved		
7. To what extent (if at all) have you worked with your team to ensure that they understand their role in the delivery of pharmacist prescribing at the advanced career stage?	If not, why not, if known?<br><br>Prompt: team working, changes to practice/roles etc.	
**Activation**- - participants need to collectively define the actions and procedures needed to sustain a practice		
8. What are (or may be) the effective actions and procedures required to successfully sustain the delivery of pharmacist prescribing at the advanced career stage?	If they don't have experience: Thinking about other interventions that the team have adopted, what might be the actions and procedures needed to sustain the delivery of pharmacist prescribing at the advanced career stage?	
**Collective action**<br>*Refers to the "operational work" required to enact a new set of practices, such as staff resourcing, equipment availability and other issues specific to local context.*		

**Interactional workability -** How do staff work collectively to get the work done?		
9. To what extent (if at all) and how do staff* work together collaboratively to successfully deliver pharmacist prescribing at the advanced career stage?	Prompt: with *in their team/other professionals/colleagues etc.?<br><br>Prompt: impact on responsibility and accountability within the team<br><br>Prompt: impact of working together on team working / resolution	
**Relational integration** - Staffs trust of each other's work and expertise		
10. How confident or not do you feel that pharmacist prescribing at the advanced career stage is being delivered appropriately?	If they don't have experience: How confident or not do you feel that pharmacist prescribing at the advanced career stage can be delivered appropriately?<br><br>How does the individual feel? Their team? Their MDT? Their pts? What are your views, what might be the views of others?<br><br>Is it being delivered broadly across whole eligible population of advanced pharmacists?	
**Skill set workability** - How is the work distributed?		
11. To what extent does pharmacist prescribing at the advanced career stage impact on who does what in the team, if at all?	If they don't have experience: How could pharmacist prescribing at the advanced career stage impact on who does what in the team?<br><br>Prompt: training required if changes needed	

**Contextual integration** - How is the work supported?		
12. **What to what extent do resources impact, on the ability to deliver and optimise pharmacist prescribing *at the advanced career stage*?**	If they don't have experience: How could resources impact, on the ability to deliver and optimise pharmacist prescribing at the advanced career stage?<br><br>Prompt: Resource may include training, support programmes, equipment, clinic space	
**Reflexive Monitoring**<br>*Refers to the "appraisal work" in understanding how a new set of practices affects those engaging with them.*		
**Systemisation** - the work of participants collecting information to determine how effective and useful it is for them and for others		
13. How have the profession been involved in evaluating the effect and usefulness of pharmacist prescribing at the advanced career stage?	What other systems or methods of assessing practice exist in your workplace, if any? How would they fit in with assessment of pharmacist prescribing at the advanced career stage, if at all?	
**Communal appraisal** - participants work together - sometimes in formal collaborative, sometimes in informal groups to evaluate the worth of a set of practices.		
14. To what extent have others have been involved in determining how effective and useful pharmacist prescribing at the advanced career stage is?	Others could include patients/colleagues, the rest of the MDT, senior leaders etc.	
**Individual appraisal** - Participants in a new set of practices also work experientially as individuals to appraise its effects on them and the contexts in which they are set.		
15. Tell me to what extent you would continue to develop pharmacist prescribing at the advanced career stage?	What would affect your decision?	
**Reconfiguration -** appraisal work by individuals or groups may lead to attempts to redefine procedures or modify practices - and even to change the shape of a new technology itself.		

16. Do you feel any changes to practice are required to improve the delivery pharmacist prescribing at the advanced career stage? If so what would they be?	Has your practice had to be modified in any way to enable pharmacist prescribing at the advanced career stage?	
